# QSAR, Molecular Docking, MD Simulation and MMGBSA Calculations Approaches to Recognize Concealed Pharmacophoric Features Requisite for the Optimization of ALK Tyrosine Kinase Inhibitors as Anticancer Leads

**DOI:** 10.3390/molecules27154951

**Published:** 2022-08-03

**Authors:** Rahul D. Jawarkar, Praveen Sharma, Neetesh Jain, Ajaykumar Gandhi, Nobendu Mukerjee, Aamal A. Al-Mutairi, Magdi E. A. Zaki, Sami A. Al-Hussain, Abdul Samad, Vijay H. Masand, Arabinda Ghosh, Ravindra L. Bakal

**Affiliations:** 1 Faculty of Pharmacy, Oriental University, Indore 453555, Madhya Pradesh, India; praveen140581@gmail.com (P.S.); drnkjain9781@gmail.com (N.J.); 2 Department of Chemistry, Government College of Arts and Science, Aurangabad 431004, Maharashtra, India; gascajay18@gmail.com; 3 Department of Microbiology, Ramakrishna Mission Vivekananda Centenary College, Kolkata 700118, West Bengal, India; nabendu21@rkmvccrahara.org; 4 Department of Chemistry, Faculty of Science, Imam Mohammad Ibn Saud Islamic University, Riyadh 13318, Saudi Arabia; aamutairi@imamu.edu.sa (A.A.A.-M.); sahussain@imamu.edu.sa (S.A.A.-H.); 5 Department of Pharmaceutical Chemistry, Faculty of Pharmacy, Tishk International University, Erbil 44001, Kurdistan Region, Iraq; abdul.samad@tiu.edu.iq; 6 Department of Chemistry, Vidyabharati Mahavidyalalya, Camp Road, Amravati 444602, Maharashtra, India; vijaymasand@gmail.com; 7 Microbiology Division, Department of Botany, Gauhati University, Guwahati 781014, Assam, India; dra.ghosh@gauhati.ac.in; 8 Department of Medicinal Chemistry, Dr. Rajendra Gode Institute of Pharmacy, University-Mardi Road, Amravati 444603, Maharashtra, India; rlbakal@gmail.com

**Keywords:** ALK tyrosine kinase inhibitors, QSAR, anticancer, molecular docking, MD simulation, MMGBSA

## Abstract

ALK tyrosine kinase ALK TK is an important target in the development of anticancer drugs. In the present work, we have performed a QSAR analysis on a dataset of 224 molecules in order to quickly predict anticancer activity on query compounds. Double cross validation assigns an upward plunge to the genetic algorithm–multi linear regression (GA-MLR) based on robust univariate and multivariate QSAR models with high statistical performance reflected in various parameters like, fitting parameters; R^2^ = 0.69–0.87, F = 403.46–292.11, etc., internal validation parameters; Q^2^_LOO_ = 0.69–0.86, Q^2^_LMO_ = 0.69–0.86, CCC_cv_ = 0.82–0.93, etc., or external validation parameters Q^2^_F1_ = 0.64–0.82, Q^2^_F2_ = 0.63–0.82, Q^2^_F3_ = 0.65–0.81, R^2^_ext_ = 0.65–0.83 including RMSE_tr_ < RMSE_cv_. The present QSAR evaluation successfully identified certain distinct structural features responsible for ALK TK inhibitory potency, such as planar Nitrogen within four bonds from the Nitrogen atom, Fluorine atom within five bonds beside the non-ring Oxygen atom, lipophilic atoms within two bonds from the ring Carbon atoms. Molecular docking, MD simulation, and MMGBSA computation results are in consensus with and complementary to the QSAR evaluations. As a result, the current study assists medicinal chemists in prioritizing compounds for experimental detection of anticancer activity, as well as their optimization towards more potent ALK tyrosine kinase inhibitor.

## 1. Introduction

The cancer kinome is currently acknowledged as a powerful target for the treatment of cancer; it comprises over 500 protein kinases, however only a few of them possess therapeutic activity. The term ALK was coined from a chromosomal rearrangement inside anaplastic large cell lymphoma (ALCL) that was described as a front companion but discovered in 1994 [1]. Anaplastic lymphoma kinase (ALK) is a kind of oncogenic protein that is often expressed in the brain, small intestine, or testis but not in normal lymphoid cells [2]. The main physiologic aspect inhibited by the anaplastic lymphoma kinase (ALK) gene is brain development, which can keep many cancers altered, including non-small-cell lung cancer (NSCLC) or anaplastic large cell lymphomas (ALCL) [3].

Moreover, ALK gene activation appears to contribute to the initiation of carcinogenesis in a variety of human cancers such as anaplastic large cell lymphoma, lung cancer, inflammatory myofibroblastic tumors, and neuroblastoma, with the end result of fusion with additional oncogenes (NPM, EML4, TIM, etc.) and gene amplification, mutation, and protein overexpression [4]. As a basis for the researchers’ involvement with the tyrosine kinase as a specific target in cancer treatment, the ALK fusion protein was created. TK plays a major role in signal transduction and is classified as a protein kinase up to the point where it is separated from the phosphate group by a tyrosine residue [5]. ALK is a recognized molecular target in a variety of ALK mutated malignancies, including non–small cell lung cancer. On the other hand, the rise of drug resistance has almost completely restricted the scientific advantage of targeting ALK with tyrosine kinase inhibitors (TI) [6]. Furthermore, because of the treatment of ALK rearranged cancer, ALK has been suggested as a therapeutic target protein.

**Figure 1:**
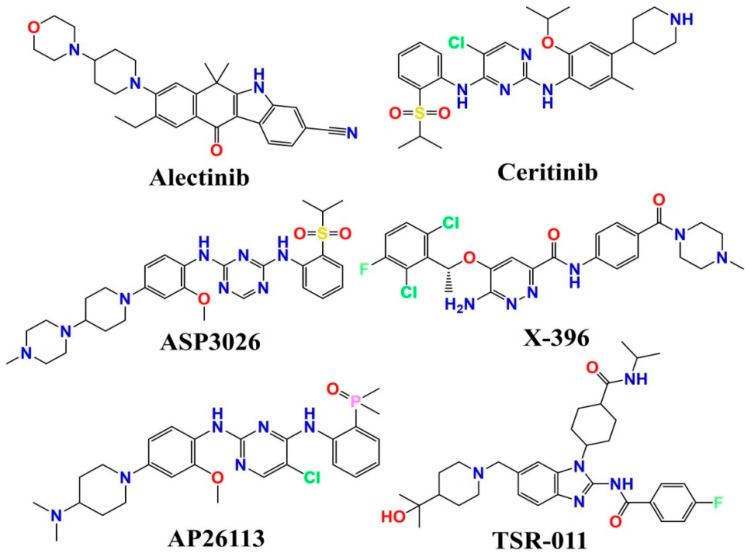
Demonstration of some ALK Tyrosine kinase inhibitors presently under clinical trials.

Academic institutions and the pharmaceutical sector are working hard to develop potent ALK inhibitors. Currently, the US Food and Drug Administration (US. FDA) has approved crizotinib, Entrectinib, ceritinib, or alectinib for the treatment of patients with metastatic “ALK-positive” NSCLC [7,8,9,10,11,12]. Small-molecular inhibitors of ALK, such as AP26113 [13] and lorlatinib (PF-06463922) [14], are currently being evaluated in clinical studies (See Figure 1). On the other hand, irreversible drug resistance is rapidly spreading over the world, endangering the efficacy of chemotherapy containing these drugs. Alectinib [2], ceritinib [3], ASP3026 [4], PF-0643922, X-396, AP26113, and TSR-011 are examples of small molecules of ALK inhibitors that have been developed and are now being tested in clinical studies) [15,16,17].

We conducted a quantitative structure activity relationship (QSAR) investigation on a dataset of 224 compounds, including clinically installed ALK tyrosine kinase inhibitory activity (Ki), in order to evaluate the critical structural and physicochemical requirements for ALK inhibitors as potent anticancer agents.

Following are the most common QSAR modelling steps: (I) selection of a dataset of molecules that cover a wide chemical space along with verified bio-activity expressed in terms of Ki, IC_50_ or EC_50_; (II) generation of 3D-structures of the molecules followed by their optimization using appropriate molecular mechanics; (III) molecular descriptor calculation and data pruning using an acceptable statistical method, if required; (IV) QSAR model development using an appropriate technique that recommends promising molecular descriptor selection; and (V) double cross validation of the developed QSAR models. Statistical QSAR evaluates the bioactivity of a chemical based on its in vitro modification and in vivo testing in a wet lab. Furthermore, illustrative QSAR legitimately vary along with the statistical parameters between QSAR models that provide deep understanding for the pharmacokinetic then optimization of the lead drug [18,19,20,21,22,23,24,25,26].

Therefore, in the present study, we have attempted to create a QSAR model by utilizing a dataset of 224 structurally diverse compounds whose ALK tyrosine kinase inhibitory activity was previously determined experimentally (Ki). Additionally, the most active compounds in the current dataset have been examined for molecular docking studies, which have been followed by MD simulation and MMGBSA calculations. The primary goal of the current work is to uncover the array of Pharmacophoric characteristics involved in the binding affinity or stability of the drug-ALK complex. Furthermore, the stability on the drug receptor complex was observed and analyzed using MD simulation or MMGBSA techniques. The QSAR model developed in this work should provide useful information to the synthetic chemists in the discovery and development of leads to more powerful ALK Tyrosine kinase inhibitors.

## 2. Results

All the statistical parameters associated with fitting, double validation, or Y-scrambling for the generation of de-novo-QSAR models 1.1–1.2, along with the respective threshold values for half of the parameters are displayed in Table 1 (at the bottom of the table).

**Figure 2:**
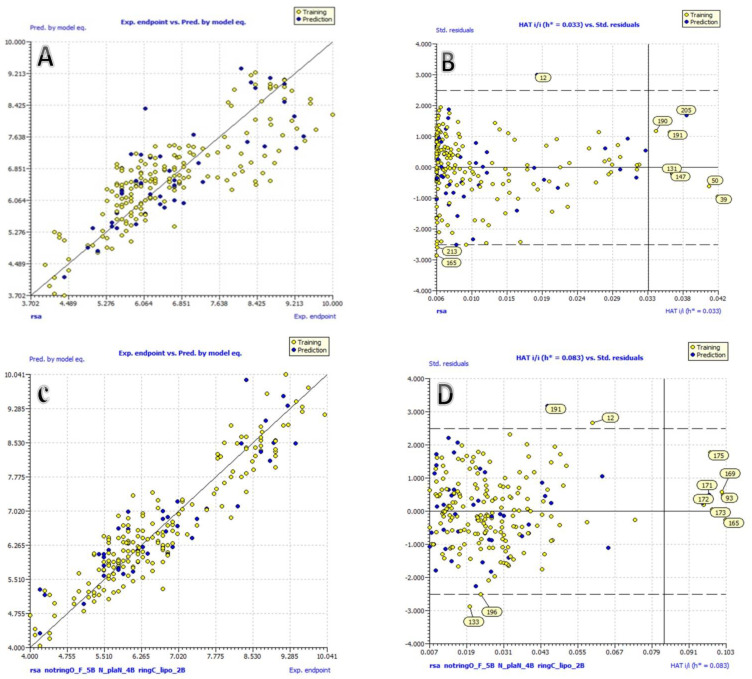
(**A**) Graph of experimental vs. Predicted pKi values for model 1.1; (**B**) Williams plot for model 1.1; (**C**) Graph of experimental vs. Predicted pKi values for model 1.2; (**D**) Williams plot for model 1.2.

Fitting parameters such as R^2^, R^2^_adj_, and CCC_tr_, among others, have achieved values well above the mentioned threshold limits, indicating the statistical acceptability of comprehensive QSAR models with a wide variety of chemical descriptors in them. Q^2^_LOO_, Q^2^_LMO_, and so on are internal validation parameters whose values indicate the statistical robustness of QSAR models. High values for external validation parameters R^2^_ext_, Q^2^-_Fn_, and so on indicate external predictability for both models which is reflected in a graph of experimental endpoint verses model predicted endpoint (Figure 2A,C). Williams plots that support applicability domain (AD) on the developed QSAR models are shown in Figure 2. To exclude the possibility of occasional improvement in QSAR models, appropriate threshold values based on sufficient parameters and minimal correlation among molecular descriptors must be maintained (See Supplementary Materials File S5 for the detailed formulas for the calculation of various QSAR model performance parameters). Statistical robustness and high external predictability are strong arguments in favor of these models.

### 2.1. Model. 1.1 (Univariate Analysis)

The univariate statistical analysis of the divided dataset QSAR model revealed that the dataset molecules have a gradual then spectacular outset (R^2^ = 0.690) with the descriptor rsa, which stands for ratio of surface area (ratio of molecular surface area to the solvent accessible surface area). The developed univariate QSAR model is as follow:

pKi = −8.9 (±1.5) + 25.0 (±2.4) × rsa(1)

### 2.2. Model. 1.2 (Multivariate Analysis)

The another QSAR model with multiple varioable is given below;

pKi = −6.7 (±1.09) + 21.6 (±2.1) × rsa + 1.08 (±0.16) × notringO_F_5B + 0.06 (±0.03) × N_plaN_4B + −0.03 (±0.02) × ringC_lipo_2B(2)

(Supplementary Materials Tables S3 and S4.)

## 3. Discussion

A precisely validated correlation between visible features of molecules, as embodied by molecular descriptors and their ALK TK inhibitory potency, amplifies the records about mechanistic features of molecules, as well as specificity and volume (presence and even absence) of various structural characteristics for favorable anticancer activity. In a broad sense, the ALK TK inhibitory efficacy of the compounds in the current dataset is the aggregate of four chemical descriptors that emerged in the developed univariate and multivariate QSAR models. Molecular descriptors may be classified into two groups based on their sign between sophisticated QSAR models.

The molecular descriptors rsa, notringO_F_ 5B, and N_plaN_4B performed well in the established QSAR models. Amplification of the values of these chemical descriptors can also contribute to an increase in the compound’s ALK TK inhibitory efficacy.

The raised QSAR models include the molecular descriptor ringC_lipo_2B with a negative coefficient, and by decreasing the value of that molecular descriptor the compound’s ALK TK inhibitory efficacy may be increased. The value of these four molecular descriptors is highlighted in the next section by comparing the variation in ALK TK inhibitory potency of the molecules (expressed in terms of Ki and pKi) with the shift in the values of the molecular descriptors seen in the QSAR models. However, the compound’s bioactivity is the result of the combined action of several molecular descriptors that may or may not have been included in QSAR models.

### 3.1. Mechanistic Interpretation

#### 3.1.1. rsa

**Figure 3:**
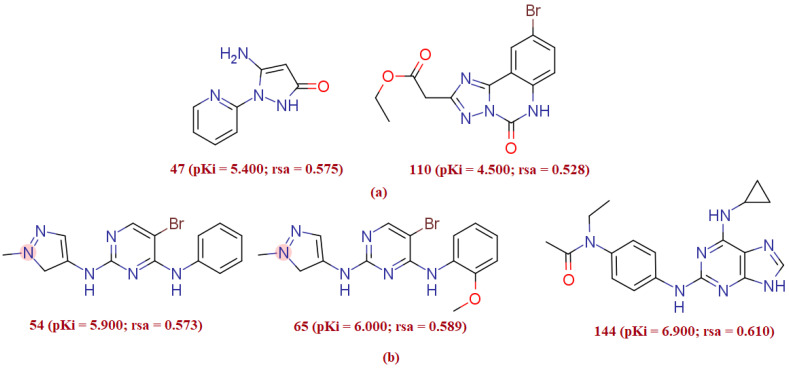
Illustration of the molecular descriptor rsa for the molecular pair 47 and 110 (**a**), and for the molecular pair; 54, 65 and 144 (**b**) only.

The present QSAR evaluation performed on a given dataset marked the ratio of molecular surface area (All_MSA, molecular surface area) to the solvent accessible surface area (All_SASA) encoded by rsa (ratio of surface area) as one of the best performing molecular descriptors with a positive relationship with ALK TK inhibitory potency of the molecule. The chemical descriptor rsa (ratio of surface area) encrypts information on the molecular surface area (All_MSA: molecular surface area of the molecule (all atoms)) to the solvent accessible surface area (All_SASA: solvent accessible surface area of the molecule (all atoms)) ratio, and has a positive relationship with the molecule’s ALK TK inhibitory potency. A small change in rsa results in a big change in the inhibitory activity of ALK TK. Because rsa is the ratio of the value of All_MSA to the value of All_SASA, the large possible value of All_SASA to the small value of All_SASA will set the rsa to the larger value, thus increasing the molecule’s ALK tyrosine kinase inhibitory efficacy. This is demonstrated by comparing molecule 178 (pKi = 10, rsa = 0.68, All_MSA = 400.5, All_SASA = 587.6) to molecule 181 (pKi = 9.252, rsa = 0.66, All_MSA = 426.6, All_SASA = 639.9). The significance of rsa may be demonstrated by another pair of molecules 110 (pKi = 4.5, rsa = 0.528) and 47 (pKi = 5.4, rsa = 0.575) that also corroborate the observation (see Figure 3a). A triad of compound 54 (pKi = 5.9, rsa = 0.573), compound 65 (pKi = 6.0, rsa = 0.589), and compound 144 (pKi = 6.9, rsa = 0.610) also highlights the importance of high value of ratio of surface area (see Figure 3b).

#### 3.1.2. N_plaN_4B

The molecular descriptor N_plaN_4B represents the number of Nitrogen atoms with four bonds from the planar Nitrogen atom and it has a positive coefficient in the developed QSAR model. A significant number of such booster pairs of Nitrogen and planar Nitrogen may provide a more powerful ALK TK inhibitory activity. This observation is reinforced by comparing the molecule 92 (pKi = 6.8, N_plaN_4B = 5) with five booster Nitrogen pairs to the molecule 78 (pKi = 5.9, N_plaN_4B = 2) with just two booster Nitrogen pairs.

**Figure 4:**
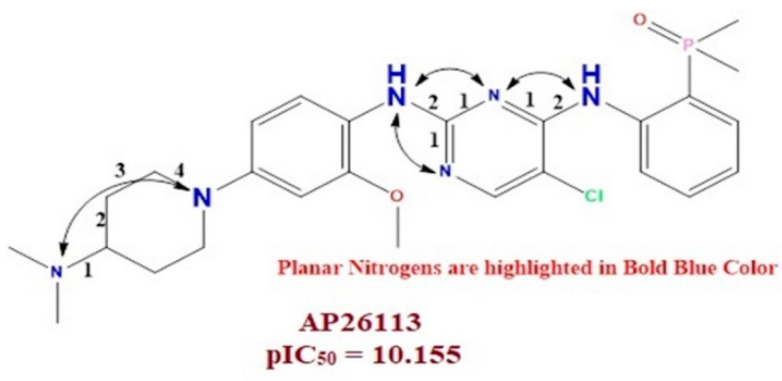
Illustration of Molecular descriptor N_plaN_4B in clinical trial molecule AP26113 (IC_50_ = 0.07 nM).

Moreover, in clinical trial agent AP26113 has such booster pairs, i.e. planar Nitrogen within four bonds from the Nitrogen atoms. The present observation confirmed that the QSAR model has successfully identified similar Pharmacophoric traits which are also present in clinical trial agents AP26113. Therefore, the planar Nitrogen within 4 bonds from the Nitrogen atom is mandatory for enhancing the affinity of ALK tyrosine kinase inhibitors (see Figure 4).

Additionally, replacement of the molecular descriptor N_plaN_4B with fplaNN4B (represent the frequency of occurrence of Nitrogen atom exactly at four bonds from the planer Nitrogen atom) (Q^2^_loo_ = 0.85, R^2^ = 0.85) and ringN_plaN_6B (occurrence of planar Nitrogen within six bonds from the ring Nitrogen atom) (Q^2^_loo_ = 0.85, R^2^ = 0.85) led to the diminution in the statistical presentation of the model. Thus, it can be concluded that the molecular descriptor N_plaN_4B is the better choice for predicting the ALK TK inhibitory potency. Consequently, the optimal value of distance between planar nitrogen and nitrogen atom is four bonds.

#### 3.1.3. notringO_F_5B

**Figure 5:**
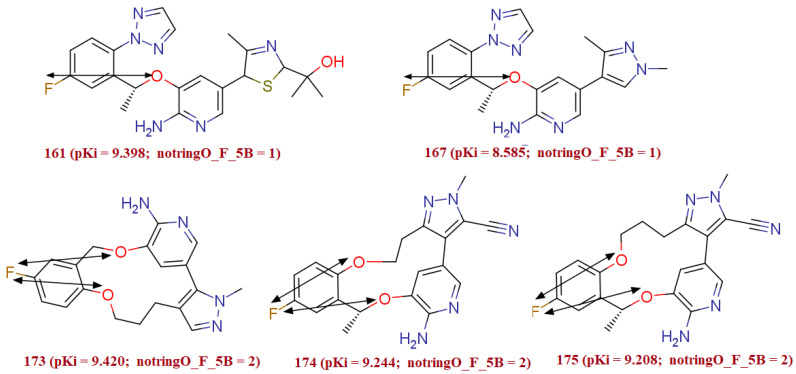
Illustration of molecular descriptor notringO_F_5B for the molecules 161 and 173, 174 and 175, and 167.

The molecular descriptor notringO_F_5B represents the number of Fluorine atoms within five bonds from the non-ring Oxygen atom. This molecular descriptor has a positive relationship with the ALK TK inhibitory activity of the compound, and therefore augmenting its value could offer a more potent ALK TK inhibitor. The significance of the presence and large value of a pair of Fluorine within five bonds from non-ring Oxygen can be rationalized from the fact that in the present dataset, the relatively least active compounds with pKi ≥ 7.400 (with very few exceptions) either Fluorine atoms itself absent or such booster pair of Fluorine and non-ring Oxygen is absent, i.e. notringO_F_5B = 0. Whereas, in most active compounds with pKi ≥ 9.155 at least one such Fluorine is five bonds away from the non-ring Oxygen atom (notringO_F_5B ≥ 1). In addition to this, there are 36 such diverse sets of compounds in the entire dataset which comprises one to two such a pair of oxygen atom and fluorine atom present within five bonds. Moreover, the compounds such as, 174 (pKi = 9.24, notringO_F_5B = 2), 175 pKi = 9.20, notringO_F_5B = 2), 165, and 167 were present in prediction set while; the rest of 32 active compounds were exist in training set. Around 16% of the molecule comprises this molecular descriptor. The occurrence of the molecular descriptor notringO_F_5B was not only limited to the series of homologues molecules, but it occurs in the diverse set of molecules like 161 and 167 also. Additional evidence in support is the molecule 161 (pKi = 9.398, notringO_F_5B = 1) with the molecule 173 (pKi = 9.420, notringO_F_5B = 2) (see Figure 5).

From this observation it is revealed that the combination of Fluorine atom with non-ring Oxygen atom is independently important for inhibitory potency of ALK TK; but shifting a fluorine atom with any sulfur atom [i.e., notringO_S_5B (Q^2^_LOO_ = 0.7219, R^2^ = 0.7344) that represent the occurrence of Sulfur atom within five bonds from the non-ring Oxygen atom] or any acceptor atom [i.e., notringO_Acc_5B (Q^2^_LOO_ = 0.7244, R^2^ = 0.7350) that represent the occurrence of acceptor atom within five bonds from the non-ring Oxygen atom] significantly diminishes the statistical presentation of the QSAR model. Therefore, the presence of a fluorine atom has good correlation with the Ki value.

#### 3.1.4. ringC_lipo_2B

**Figure 6:**
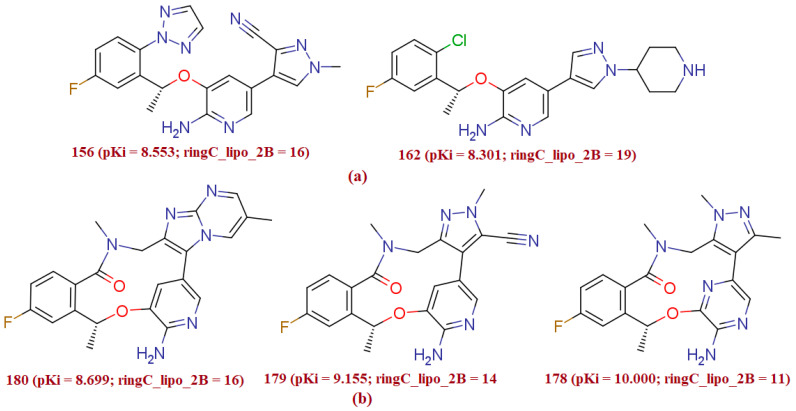
Presentation of the molecular descriptor ringC_lipo_2B for the molecular pair 156 and 162 (**a**) and for another molecular pair; 180, 179 and 178 (**b**) only (the ring carbon atoms within 2 bonds from the lipophilic carbon atoms are highlighted by blue ball).

The molecular descriptor ringC_lipo_2B encodes information on the occurrence of the ring carbon atoms within two bonds from lipophilic atoms. This observation is supported by comparing the pKi value of the molecule 156 (pKi = 8.55, ringC_lipo_2B = 14) with the molecule 162 (pKi = 8.30, ringC_lipo_2B = 19), for which decrease in the value of the molecular descriptor ringC_lipo_2B for the molecule 162 to 14 resulted into an increase in the pKi value by about 0.25 per unit. The triad of the molecules 180 (pKi = 8.699, ringC_lipo_2B = 16), 179 (pKi = 9.155, ringC_lipo_2B = 14), 178 (pKi = 10, ringC_lipo_2B = 11) also signifies the importance of the molecular with this Pharmacophoric future (see Figure 6a,b). This is obvious as the macrolides and aromatic rings are quite abundant in the present dataset molecules.

On the other hand, when we have shifted molecular descriptor ringC_lipo_2B with the descriptors ringC_lipo_1B and fringCnotringC1B in which the statistical performance of the QSAR model was meaningfully improved with the molecular descriptor fringCnotringC1B (Q^2^_LOO_ = 0.87, R^2^ = 0.87); while performance slightly goes down with the descriptor ringC_lipo_1B (Q^2^_LOO_ = 0.84, R^2^ = 0.85). Therefore, from the present observation, it is revealed that with an increase in numbers of the non-ring carbon atoms attached directly to the ring carbon atoms, TK inhibitory potency could increase. Based on this observation, the optimal distance between non ring carbon atom/lipophilic atom and ring carbon atom must be one. Moreover, we have highlighted the structure of molecule 156 and 162 to rationalize the impact of the molecular descriptor ringC_lipo_2B. Absence of the triazole ring, methyl group on the pyrazole ring and carbonitrile group significantly affects the TK inhibitory potency, and may be the possible reason for the decline in the potency of the molecule 162.

### 3.2. Molecular Docking

ALK was discovered to be a new receptor tyrosine kinase (RTK) with an external ligand-binding domain (1030 amino acids), a transmembrane domain (28 amino acids), and an intracellular tyrosine kinase domain based on the amino acid sequences (561 amino acids) [26,27]. While the human ALK tyrosine kinase domain is very comparable to the insulin receptor, its extracellular domain is unique among the RTK family in that it comprises two MAM domains (meprin, A5 protein, as well as receptor protein tyrosine phosphatase mu), an LDLa domain (low-density lipoprotein receptor class A), and a glycine-rich region [27,28].

ALK’s ATP binding site has 27 residues, and to boost selectivity against other kinases, residues that differ from ALK were targeted. The ALK Leu1198 residue is preserved in 26 percent of the kinome and is typically Phe or Tyr in other kinases. By expanding into this pocket and bumping against the bigger Phe and Tyr residues, this smaller Leu residue might potentially provide selectivity against the majority of kinases (60 percent) [14].

**Figure 7:**
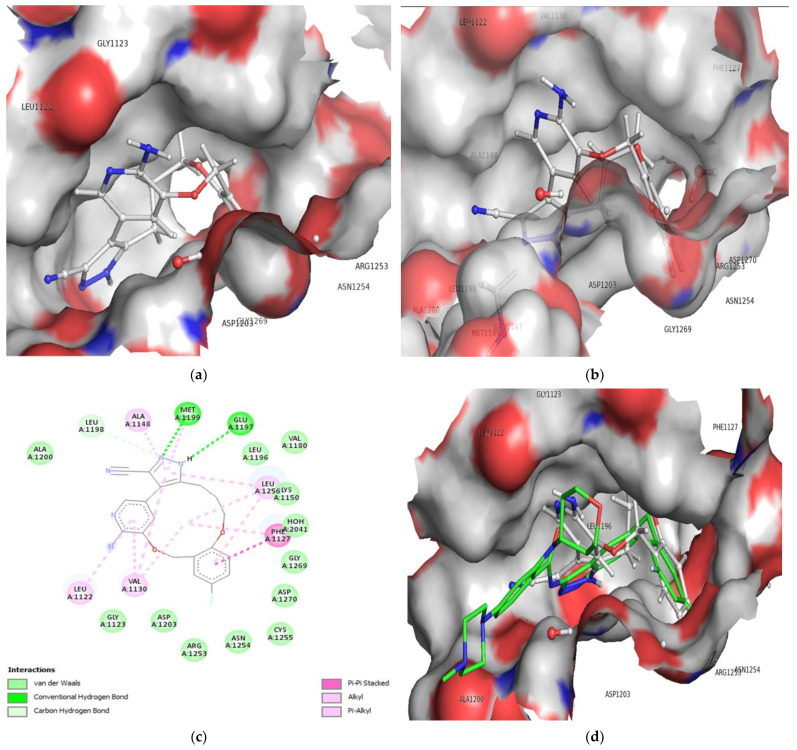
Depiction of alignment of compound 172 within the active site of ALK tyrosine kinase (**a**,**b**), 2D interaction of compound 172 with ALK tyrosine kinase (**c**) and Superimposed conformation of compound 172 with the pdb 5fto ligand (green colored); Entrectinib (**d**).

The protein data bank provided the ALK tyrosine kinase pdb file (pdb id-5fto, Resolution 1.7 Å). The pdb 5fto was chosen for its X-ray resolution or sequel completeness. The protein 5ft was prepared by UCF Chimera chimera-1.16-win64 software (https://www.cgl.ucsf.edu/Visitors/index.html, Oakland, California, accessed on 2 March 2022). During protein preparation, we have retained water molecules. High affinity for a protein target must be attained as it is a crucial component of drug design. Although there are statistical mechanics-based formal mathematical equations that can be used to calculate binding free energies, doing so in practice is quite challenging, especially when the effect is caused by a single water molecule rather than the bulk properties of water and it is impossible to capture solvation effects. It is impossible to avoid these granular effects; a review of PDB structures reveals that each ligand-protein combination contains 4–6 ligand-bound water molecules. Furthermore, water not only stabilizes ligand interactions but plays a biological role in dictating specificity. Therefore, the improved protein with water molecules was appropriate for docking analysis. Before the docking investigation, the natural ligand (Entrectinib) was removed; in the present study, the binding site for native ligand, namely the active site, has been studied. As a result, the compounds were docked between the active site, where the native ligand was originally bound orthosterically along ALK tyrosine kinase, and the docking pose for the most active molecules 172 and 178, and an example is shown here for convenience (see Figure 7a,b). Based on the activity profile, we have carried out molecular docking analysis of the compounds 172 and 178 only. The docking analysis of compound 172 into the ALK tyrosine kinase binding pocket revealed conventional hydrogen bonding, carbon hydrogen bonding, pi-pi stacked, and pi-alkyl hydrophobic interactions (See Figure 7c), with a docking score of −8.009 kcal/mol (RMSD = 0.84 Å) and binding energy of −77.42 kcal/mol (see Table 2). In the binding pocket of the ALK tyrosine kinase, compound 172 adopts the same collapsed conformation as the co-crystallized ligand Entrectinib (See Figure 7d). The hydrogen atom on the N1 nitrogen between the pyrazole ring performs conventional hydrogen bonding with the oxygen atom of the residue GLU1197 forming the hinge region with the interatomic association of 2.87 Å, where the oxygen atom of the stated residue acted as hydrogen bond acceptor, and the hydrogen atom on the N1 nitrogen atom acted as hydrogen bond donor. Alongside, another conventional hydrogen bond was discovered in the hydrogen atom on the N2 pyrazole nitrogen with the residue MET1199 of the hinge region (interatomic distance 2.17 Å), within which pyrazole nitrogen appeared as like a hydrogen bond acceptor along MET1199 residue emerged as a hydrogen bond donor in the current composite 172-ALK tyrosine kinase complex. Table 2 displays the full docking results for the composite 172. Furthermore, the gatekeeper residue LEU1198 was attached with N2 nitrogen over the pyrazole ring through carbon hydrogen bonding along an interatomic distance of 2.82 Å. It was reported that the LEU1198 decide the selectivity of the ligand against variety of the kinases. The present observation in the docking analysis supports this fact.

Moreover, at an interatomic distance of 4.81 Å, the pi-pi stacking hydrophobic contact has been facilitated by the indulgence of the pi orbital of the benzene ring and the pi orbital of the PHE1127. Furthermore, in compound 172, the pyrimidine ring is anchored with VAL1130 (interatomic distance 4.28 Å) and LEU1256 (interatomic distance 4.56 Å) and is coupled to the benzene cyclononaphane ring by alkyl hydrophobic contact. VAL1130 (interatomic distance 4.48 Å) and LEU1256 (interatomic distance 5.07 Å) make pi-alkyl hydrophobic contact with the pyrimidine ring and the cyclononaphane ring at the same time. Furthermore, the presence of an ether linkage in the unsaturated cyclononaphane ring amplifies the hydrophobicity of compound 172 as compared to the saturated benzene ring and, to a lesser extent, pyrimidine ring carbons. These findings validate the significance of the cyclononaphane ring in the molecule 172, which is primarily responsible for the compound’s efficacy as mediated by the hydrophobic contact with the ALK tyrosine kinase.

As a result, it can be concluded that the molecule 172 that binds to the ALK tyrosine kinase and drug receptor complex was mostly stabilized via conventional hydrogen, carbon hydrogen, pi-pi cation contact, alkyl hydrophobic and pi-alkyl hydrophobic interactions (See Figure 7).

Moreover, the residues: ALA1148 (interatomic distance 3.54 Å), MET1199 (interatomic distance 5.43 Å), LEU1256 (interatomic distance 4.62 Å), LEU1122 (interatomic distance 4.93 Å), VAL1130 (interatomic distance 3.94 Å) and LEU1256 forming part of a glycine rich loop (interatomic distance 4.17 Å) establishes a pi-alkyl hydrophobic interaction with the pi electrons of the pyrimidine and benzene rings, strengthening the molecule 172-ALK tyrosine receptor complex.

**Figure 8:**
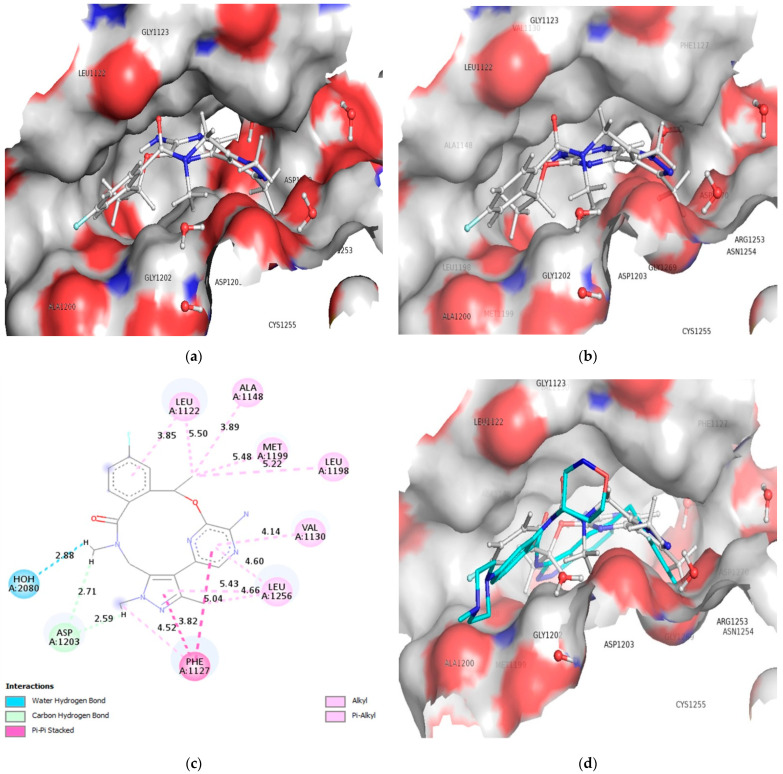
Depiction of alignment of compound 178 within the active site of ALK tyrosine kinase (**a**,**b**), 2D interaction of compound 178 with ALK tyrosine kinase (**c**), and Superimposed conformation of compound 178 with the pdb-5fto ligand (cyan colored): Entrectinib (**d**).

Furthermore, the docking analyses for compound 178 reveal the stability of the drug receptor complex through the formation of water-mediated hydrogen bonds, carbon hydrogen bonds, pi-pi stacking hydrophobic contacts, alkyl and pi-alkyl interactions, and a binding energy of −87.50 kcal/mol (docking score −7.84 kcal/mol, RMSD: 1.06 Å). The HOH2080 water molecules display hydrogen bonding contact with the N7 nitrogen atom of the cyclononaphane ring with an interatomic distance of 2.87 Å (see Figure 8a,b). At the same time, hydrogen of the N7 nitrogen produced by keto-enol tautomerism binds to the ASP1203 residue (interatomic distance 2.58 Å). This interaction is mediated by the presence of N7 nitrogen as a hydrogen bond donor and the oxygen atom of the ASP1203 residue. In addition, ASP1203 and the N1 nitrogen atom of the pyrazine ring in compound 178 formed another carbon hydrogen bond (see Figure 8a,b) (see Table 3). The superimposed conformation of compound 178 with the pdb-5fto ligand Entrectinib into the binding pocket of ALK TK is shown in Figure 8c,d.

**Figure 9:**
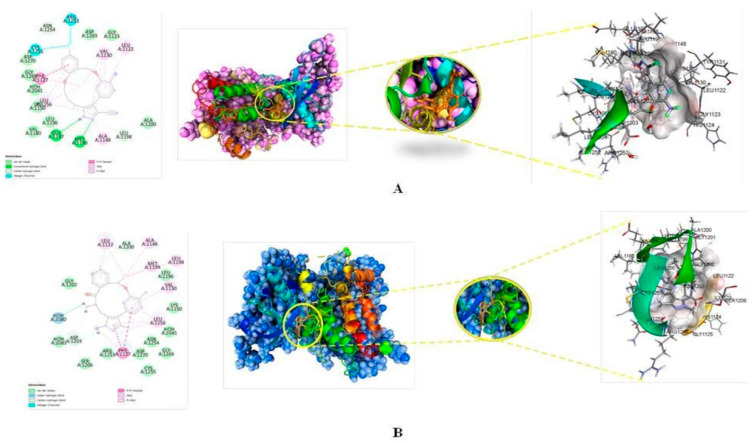
(**A**) Best docked pose of 172 with ALK displaying 2D interaction plot on the left panel. Pink dashed lines indicating the Pi-Alkyl bond and residues embedded in light green sphere indicating to involve in Van der Waals interactions. On the center panel, surface view of ALK displaying binding cavity of 172 and right panel displaying the zoomed out binding pocket having amino acid residues surrounding the 172 molecule; (**B**) Best docked pose of 178 with ALK displaying 2D interaction plot on the left panel. Pink dashed lines indicating the Pi-Alkyl bond and residues embedded in light green sphere indicate involvement in Van der Waals interactions. On the center panel, surface view of ALK displaying binding cavity of 178 and right panel displaying the zoomed out binding pocket having amino acid residues surrounding the 178 molecules.

Interestingly, the two pi-pi stacking hydrophobic contact into the drug receptor complex has been sustained and facilitated by the involvement of pi electrons from the pyrazine ring, pyrimidine ring, as well as pi electrons from the saturated benzene ring in residue A: PHE1127 (Interatomic distances 5.03 and 3.82 Å resp.). As a result, the residues A: ALA1148 (Interatomic distance 3.88 Å), A: LEU1122 (Interatomic distance 5.49 Å), A: LEU1198 (Interatomic distance 5.22 Å), and A: MET1199 (Interatomic distance 5.47 Å) establish an alkyl hydrophobic contact with the cyclononaphane ring’s C4 substituted methyl moiety. Furthermore, the pi electrons of the benzene ring at residue A: PHE1127 (interatomic distance 4.51 Å) establish pi-alkyl hydrophobic contact with the alky moieties of the pyrazine and pyrimidine rings. The pyrazine ring and pyrazine then form a two pi-alkyl contact with the residue A: LEU1256, with interatomic distances of 5.43 and 4.60 Å, respectively. Similarly, the residues A: VAL1130 and A: LEU1122 were linked with the pyrimidine ring and benzene ring via pi-alkyl hydrophobic interactions (Interatomic distances 4.14 Å and 3.84 Å, respectively). The Figure 9A,B displays the 2D interaction and surface view for the compound 172 and 178.

In case of the molecule 178, there is a complete reversal of the conformation in comparison with the reported pdb 4CMU for the molecule 178. In the present work, we have used pdb 5fto for performing docking for the most active molecules 172 and the 178. The co-crysallized native ligand was docked along the 172 and the molecule 178. The RMSD values for the molecule 178 was found to be 1.06 Å, while co-crystallized ligand (pdb-5fto) displayed a RMSD of 1.19 Å, which was less than the molecule 178. This observation revealed the good fit of the molecule 178 into the binding pocket of the ALK tyrosine kinase due to the relatively higher flexibility of the ligand. The success rates in binding mode prediction for different docking programs such as, AutoDock 4 version (v4.2.6), FlexX 1.8, FRED (OEDocking 4.1.2.1), Glide 6.7, CCDC GOLD Suite 5.3, and ICM-Pro docking on the numerous known ligands when the RMSD cutoff ranges from 1.0 to 3.0 Å [29]. The same docked complex of the molecule 178 with ALK TK was analysed for the stability by MD simulation and MMGBSA. Although most of the existing docking programs were developed as a general methodology for different systems, they do have their own strengths and limitations and may show different performances on specific applications.

The simulation studies revealed the stability of 178 into the binding pocket of ALK TK, although it displayed reverse conformation. Moreover, the reversal of the conformation could also be attributed to the large size of active site of ALK TK (See Figure 8a,b), which allows adoption of different conformations for molecule 178. Additionally, recent studies point out that current docking software like AutoDock 4 version (v4.2.6), Dock (version 3 and 6), NRG Suite (PyMOL versions 1.2 and above) etc. and respective algorithms for docking scores are inclined toward flexibility of ligands which in turn is associated with loss of ligand conformational entropy on binding. Various factors such as binding site characteristics, one-dimensional properties of the compound library, the type of the binding pocket, ligand and protein flexibility and input differences apparently decide the docking performance [30]. Therefore, the molecule 178 (docking score −7.84 kcal/mol) with lower binding affinity for ALK TK has displayed a high degree of flexibility. Thus, all these combined factors resulted in an artificially more favorable binding score for more flexible decoys than for actives.

In addition to this, when we have redock the molecule 178 again into the binding pocket of ALK TK for comparing the docking results with the QSAR findings, it has attained similar conformation as that of the co-crysallized ligand (pdb-5fto) and the molecule 172. This observation supports the reported finding related to the loss of bioactive conformation due to the high degree of flexibility.

### 3.3. Comparison of Molecular Docking Results with the Reported X-ray Evidences

**Figure 10:**
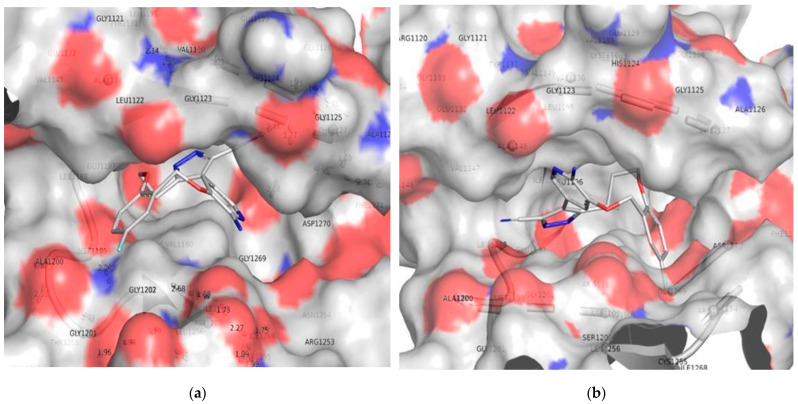
Depiction of the docking pose for the molecules 171 (**a**) and 172 (**b**) within the binding pocket of the ALK tyrosine kinase.

For comparing the docking results with the QSAR findings, it has attained similar conformation to that of the co-crysallized ligand (pdb-5fto) and the molecule 172. The docking position of molecule 172 shows that the phenyl with fluorine as a substituent is within the cavity formed by GLY1269 and ASN1254. Maria Menichincheri et al. [31] reported a similar observation. The molecule 171 has a comparable benzene ring with a fluorine substituent; however, the docking position shows that the fluorine carrying ring is unable to occupy the cavity produced by GLY1269 and ASN1254. Furthermore, conformation is completely reversed for molecules 171 and 172. One probable explanation is the existence of an extra carbon atom in molecule 172, which has resulted in increased flexibility and rsa (ratio of surface areas = ALL_MSA/ALL-SASA). As a result, QSAR and docking led to consistent and complementary results (see Figure 10).

**Figure 11:**
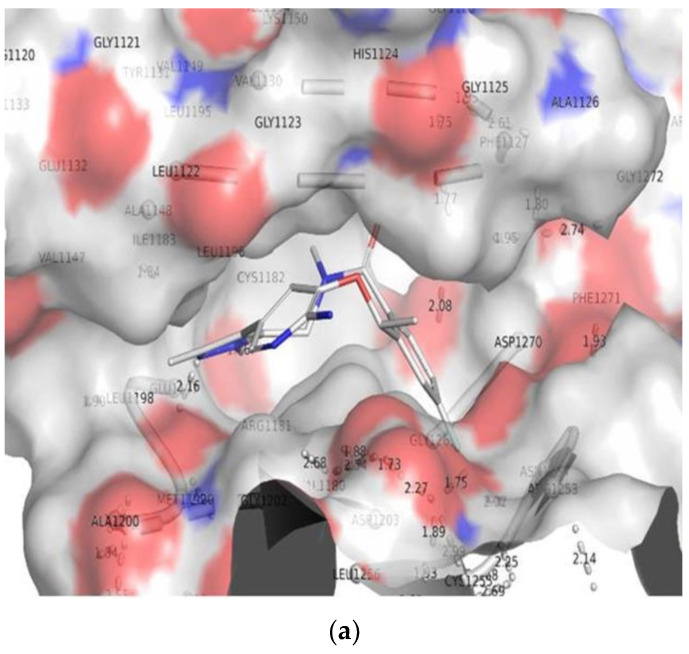

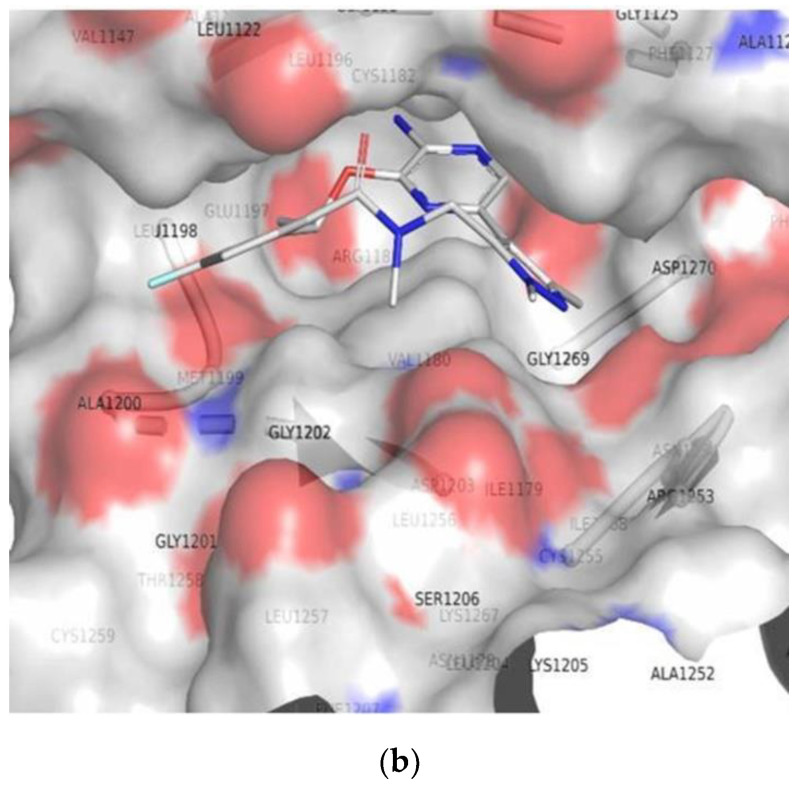
Depiction of the docking pose for the molecule 178 (**a**) and 176 (**b**) within the binding pocket of the ALK tyrosine kinase.

Similarly, comparing molecules 176 and 178 indicates an intriguing impact of the N_Plan_4B and ringC_lipo_2B on docking position and activity profile. When compared to molecule 176, molecule 178 has a larger number of N_Plan_4B and a lower value of ringC_lipo_2B. This might be the explanation for the docking conformation reversal and variances in binding affinities. The added planer nitrogen appears to be boosting the polarity of the molecule (see Figure 11a,b).

The combined effect of increase rsa and the presence of notringO_F_5B have resulted in increased potency for 218 as compared to 214. Another example is the pair of molecules 191 verses 205. This observation again divulges that the QSAR results and docking outcomes are in complete agreement with each other.

### 3.4. Molecular Dynamics Simulation (MD)

**Figure 12:**
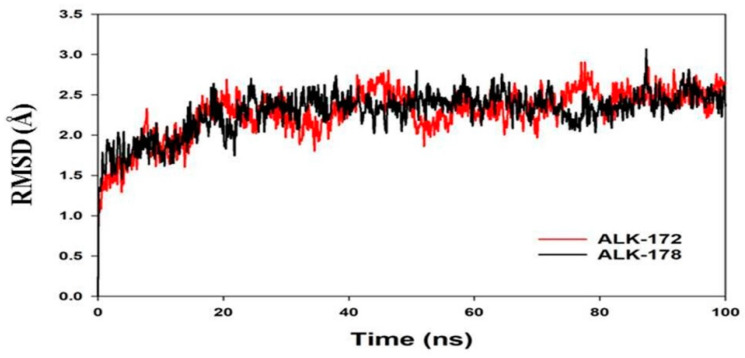
MD simulation trajectory analysis of Root Mean Square Divisions (RMSD) of 172 and 178 bound with ALK at 100 ns time frame displayed H-Bond plot of 172 bound ALK (red), 178 bound ALK (black).

Molecular dynamics and simulation (MD) experiments were performed to investigate the stability or convergence of the most active compounds 172 and 178 bound ALK complex. Based on the activity profile and molecular docking results, we have used dock complexes of the compound 172 and 178 for MD simulation analysis. When the root mean square deviation (RMSD) data were compared, each simulation including 100 ns revealed stable conformation. The C-backbone of ALK bound to 172 exhibited a deviation of about 2.2 Å (see Figure 12) while the C-backbone of ALK bound to 178 exhibited a deviation of about 1.8 Å (Figure 12); RMSD plots are within the acceptable range signifying the stability of proteins in the 172 and 178 bound state earlier than or after simulation; however, it can also be suggested that the two ligands, 172 and 178 bound to ALK is quite stable within complex.

**Figure 13:**
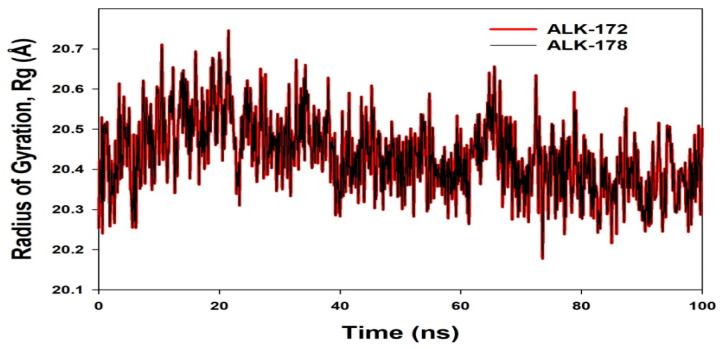
MD simulation trajectory analysis of Radius of gyration (Rg) of 172 and 178 bound with ALK at 100 ns time frame displayed H-Bond plot of 172 bound ALK (red), 178 bound ALK (black).

The radius of gyration is a measure of the protein’s compactness. The Radius of Gyration was reduced in 172 and 178 bound proteins, respectively (see Figure 13). According to the overall quality analysis based on RMSD and Rg, 172 or 178 bound to the protein targets subsequently in the binding cavities and plays a significant role in the protein stability.

**Figure 14:**
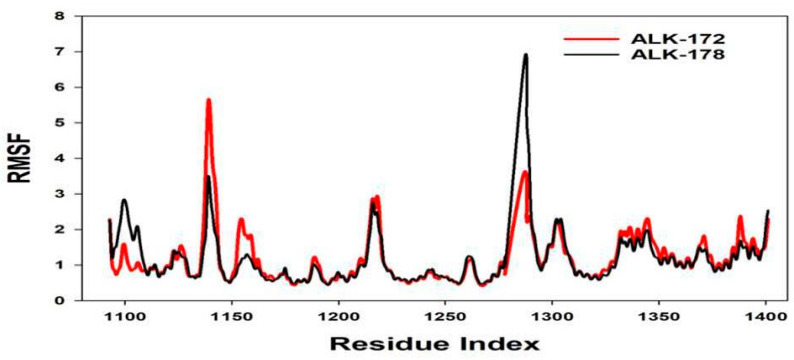
MD simulation trajectory analysis of Root Mean Square Fluctuations (RMSF) of 172 and 178 bound with ALK at 100 ns time frame displayed H-Bond plot of 172 bound ALK (red), 178 bound ALK (black).

Plots for root mean square fluctuations (RMSF) of amino acid residues are shown at a time function of 100 ns. From the 100 ns simulation runs on ALK shown in Figure 14, ligand 172 has few variations peaks at residue indices 1145, 1220, and 1290, but ligand 178 has fluctuations at residues 1139, 1220, 1275, and 1345, although it was subsequently stabilized. As a result of the RMSF plots, it is reasonable to conclude that the protein structures were stable throughout the simulation within the 172, 178 bound conformation.

**Figure 15:**
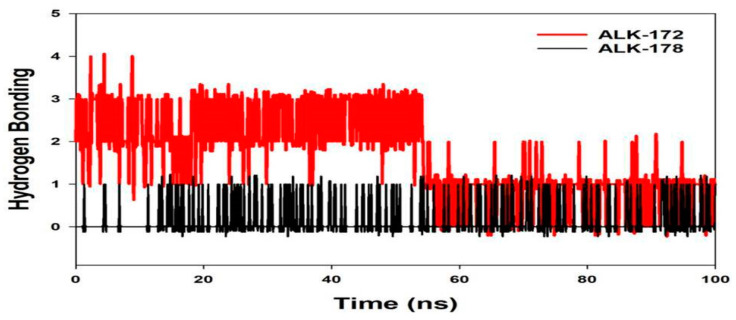
MD simulation trajectory analysis of Hydrogen Bonding (H-Bonds) of 172 and 178 bound with ALK at 100 ns time frame displayed H-Bond plot of 172 bound ALK (red), 178 bound ALK (black).

The average hydrogen bonds established in 172 and 178 or the corresponding proteins throughout the 100 ns simulation were also noticed and recorded in Figure 15. From 0 ns to a100 ns, an average of one hydrogen bonding is seen throughout the simulation or the same for MD simulations on 172 and 178 including ALK (Figure 15). Overall, three hydrogen bonds were generated during the simulation, as determined by a 2D ligand binding plot of 172 bound ALK protein, whereas in 178 bound along ALK, an average of one hydrogen bonding was produced. The quantity of hydrogen bonding over ALK along 172 and 178 hold strengthened the binding, assisting in making it more stable during the simulation (See Figure 15). In molecular docking studies, we have observed from the 2D interaction diagram where in ALK-172, we can see two hydrogen bonds were formed, on the other hand for ALK-178 we have observed single hydrogen bonding, therefore the same pattern for Molecular Dynamics.

**Figure 16:**
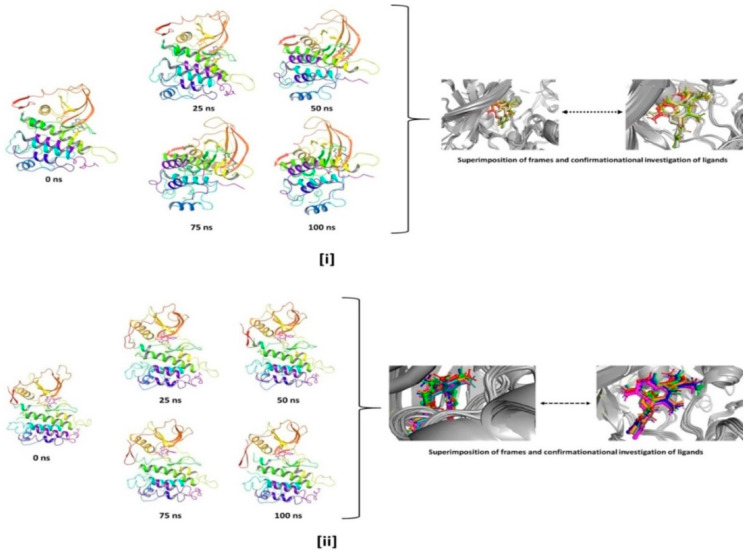
Stepwise trajectory analysis for every 25 ns displaying the protein and ligand conformation during 100 ns of simulation of [**i**] ALK-172 and [**ii**] ALK-178.

The step wise analysis of the stimulation trajectory of every 25 ns from beginning to end is depicted in Figure 16. The simulation trajectories exhibited the ligand 172 and 178 having no significant conformational changes throughout the 100 ns simulation. This signifies that the simulation complexes of ALK-172 and ALK-178 are stable and the ligand conformations at the active binding pocket of the ALK remains significantly unaltered (See Figure 16).

### 3.5. Molecular Mechanics Generalized Born and Surface Area (MMGBSA) Calculations and Energy Calculations

**Figure 17:**
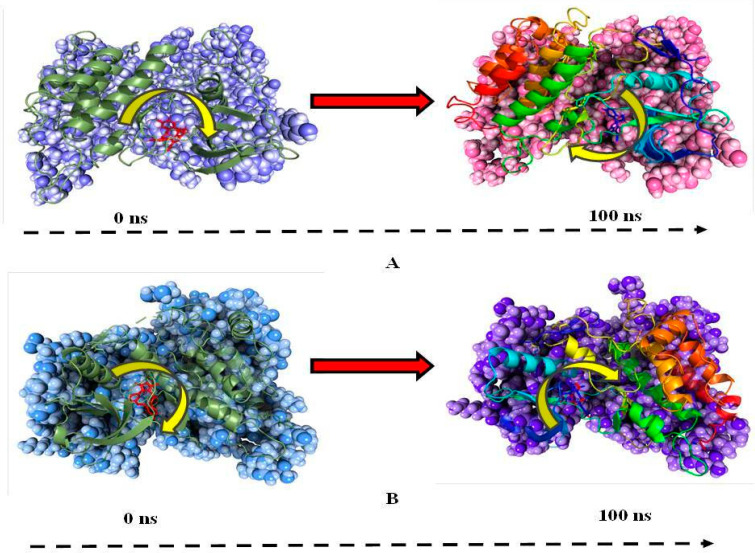
MMGBSA trajectory (0 ns, before simulation and 100 ns, after simulation) exhibited conformational changes upon binding the ligands with the protein, (**A**) ALK-172; (**B**) ALK-178. The arrows indicating the overall positional variation (movement and pose) of 172 and 178 at the binding site cavity.

The MMGBSA technique is widely used to calculate the binding energy of ligands to protein molecules. With ALK, ligand 172 has the lowest binding energy of −49.3 kcal/mol, whereas 178 has a binding energy of −52.5 kcal/mol. The GbindvdW, GbindLipo, and GbindCoulomb energies contributed the most to the common binding energy of all kinds of interactions. Gbind is governed by non-bonded interactions such as GbindCoulomb, GbindCovalent, GbindHbond, GbindLipo, GbindSolvGB, and GbindvdW. Across all interactions, the GbindvdW, GbindLipo, and GbindCoulomb energies contributed the most to the average binding energy. On the other hand, the GbindSolvGB and Gbind Covalent energies contributed the least to the final average binding energies. Furthermore, the GbindHbond interaction values of the 172-ALK and 178-ALK complexes indicated stable hydrogen bonds with amino acid residues. GbindSolvGB and GbindCovalent had negative energy contributions in all of the compounds, and so opposed binding. When coupled, GbindSolvGB and GbindCovalent verified adverse energy contributions and hence resisted binding. Figure 17 (left panel) shows that 172 and 178 at the ALK binding pocket experienced an angular shift of the angle (curved to straight) after post simulation at pre-simulation (0 ns) (100 ns) (see Figure 17). These conformational alterations result in improved binding pocket acquisition and engagement with residues, resulting in increased stability and binding energy (see Table 4).

**Figure 18:**
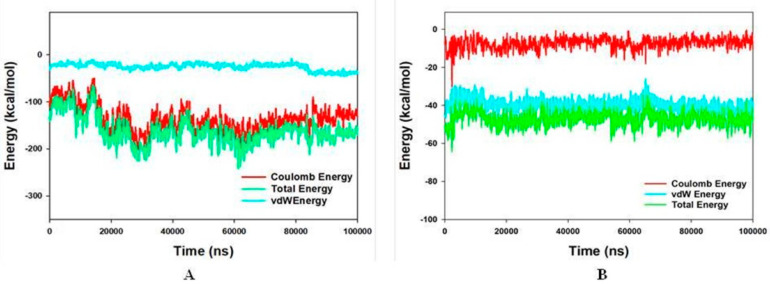
Energy plot of protein ALK with; (**A**) 172 complex system during the entire simulation event of 100 ns; (**B**) 178 complex system during the entire simulation event of 100 ns. The total energy (light green), van der Waal’s energy (cyan) and coulomb energy (red) of the entire system indicating the stability of the individual systems bound to molecule.

As a result, it is possible that the 172 (See Figure 18A) and 178 (See Figure 18B) molecules have a high affinity for the primary target ALK. In ALK bounded 172 complex systems, the average total energy was −130.00 kcal/mol (green), while van der Waal’s energy (vdW) seemed to be merged over the total energy with an average energy of −30.00 kcal/mol, which was seen as the primary contributor to the stability of the ALK172 complex (cyan). Furthermore, coulombic interactions had a little impact on system stability, contributing an average energy of −101.00 kcal/mol (red) (See Figure 18). The energy profiles of the protein, ALK, and 178 complex systems were chosen to demonstrate the overall system’s stability. In this regard, the Total Energy of the ALK-178 system has demonstrated to be completely stable, with an average total energy of −55.00 kcal/mol (dark green). However, van der Waal’s energy (vdW) remained merged up-on the total energy with an average energy of −40 kcal/mol, taking into account as the primary contributor to the ALK-178 complex’s stability (cyan). Furthermore, coulombic interactions performed a minimal influence in system stability, supplying an average energy of −10.00 kcal/mol (red) as seen in Figure 18.

**Figure 19:**
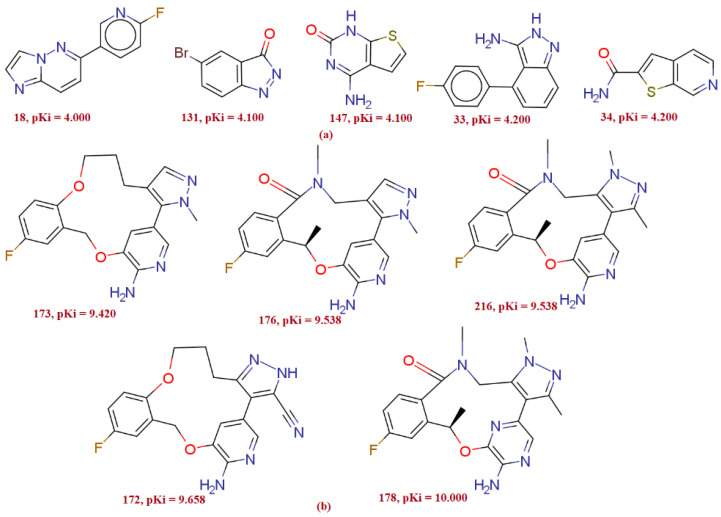
Variations in the Ki and chemical structure in the present dataset of ALK tyrosine kinase inhibitors: Five most active compounds (**b**), and five least active compounds (**a**) from the present series.

Thus, MM-GBSA calculations resulted from MD simulation trajectories that were well justified with the binding energy obtained from docking results. Furthermore, the last frame (100 ns) of MMGBSA displayed the positional change of the 172 and 178 as compared to the 0 ns trajectory, indicating the better binding pose for best fitting in the protein’s binding cavity (See Figure 19).

## 4. Materials and Methods

### 4.1. Selection of Data-Set

For the current study, 224 molecules with diverse structural features were selected due to the presence of different scaffolds and the substantial variation in the activity profile with an experimentally determined inhibition coefficient (Ki) for ALK tyrosine kinase [32,33,34,35]. Ki values ranging from 0.1 to 100,000 nM were changed to pKi (Ki = −log Ki) before actual QSAR evaluation for the ease of handling the data. The Figure 19 depicts five most active molecules followed by five least active molecules, indicating the heterogeneity of bio-activity and chemical properties. The Table 5 displays SMILES notations alongside ChEMBL id [36] and reported Ki and pKi values for several sample compounds. (See Table S1 in Supplementary Materials displaying Sr no, ChEMBL id, smiles notation, Ki value, and pKi values).

### 4.2. Molecular Structure Drawing and Optimization

The complete 224 molecules’ 2D structures were drawn using free and open source software’s ChemSketch 12 Freeware (https://www.acdlabs.com/resources/free-chemistry-software-apps/chemsketch-freeware/ accessed on 2 March 2022, version 2021), while their 3D structures were generated using Open Babel 2.4, respectively. Furthermore, optimization of the full dataset molecules was achieved using the MMFF94 force field provided in TINKER (default settings), whilst Open3DAlign was used for molecular alignment, respectively [37]. 

### 4.3. Molecular Descriptor Calculation and Objective Feature Selection (OFS):

PyDescriptor, which is available as a plugin in the PyMOL 2.5 software application, was used to calculate descriptors for each molecule [38]. Molecular descriptors with almost constant values (>95 percent) and co-linearity (|R|) greater than 0.95 were eliminated using objective feature selection (OFS) stability among QSARINS v2.2.4 [39]. This approach removed unnecessary molecular descriptors that impact multi-collinear and mock variables in the GA-MLR model. As a result, following OFS treatment, about 3339 molecular descriptors were separated in order to develop QSAR models.

### 4.4. Subjective Feature Selection, QSAR Model—Development and Validation

The condensed pool of computed molecular descriptors includes 1D- and 3D-descriptors, as well as molecular properties or value descriptors, and so on. Coinciding with a huge molecule, followed by a vivid gap. After developing robust QSAR models, the Subjective Feature Selection (SFS) function in QSARINS v2.2.4 is used to run Genetic Algorithm (GA) based multi linear regression (MLR). QSAR models were developed in accordance with OECD guidelines and were then subjected to extensive internal or external statistical validation, Y-scrambling, or Applicability domain examination. The following steps are included in the QSAR model development practice. The whole dataset was utilised to create QSAR models, which were mostly based on the undivided (training set) dataset, however in this study, we are presenting one univariate divided set and another divided set multivariate QSAR models

1. The QSAR techniques have been anticipated to use a loosely split operation in QSARINSv2 software v2.2.4 based on a divided dataset. It divided a given dataset into 80% training (180 molecules in the training set) and 20% prediction (44 molecules in prediction set). The 180 molecules from the training set were used to generate the QSAR model, and external validation was completed on 44 compounds from the prediction set.
2. The QSARINS software v2.2.4 program was used to construct GA-MLR mainly based QSAR models, incorporating default parameters. Q^2^_LOO_ is utilised as a fitness parameter to accomplish subjective feature selection. While doing SFS, the Q^2^_LOO_ value was extraordinarily prolonged up to the four variables, but an insignificant uplift was seen after that. Thus, in order to keep the QSAR model from over-fitting, SFS was previously limited to a set of four descriptors. This resulted in the creation of simple and predictive QSAR models. (See Supplementary Materials Table S2 values for the selected four molecular descriptors present in QSAR models).

**Figure 20:**
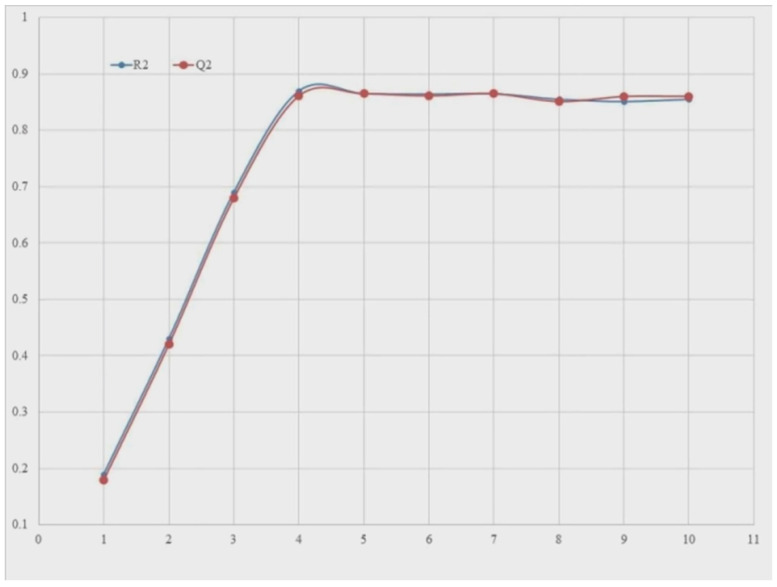
Plot of number of descriptors against Coefficient of Determination R^2^ and Leave-One-Out Coefficient of Determination Q^2^ to identify the optimum number of descriptors.

An important aspect of developing a good QSAR model with minimal over-fitting and appropriate interpretability is to have an enough number of molecular descriptors in QSAR the model. In the present study, a plan (see Figure 20) was projected in the large range of molecular descriptors included among the model yet R^2^_tr_ and Q^2^_LOO_ values to get the so-called breaking point. As a result, the variety of chemical descriptors related to the breakdown point used to be prioritized for model construction. Figure 20 shows that the breakage point correlates with four different factors. As a result, QSAR models with more than four descriptors were rejected.

To perform acceptable validation, QSARINS v2.2.4 was used to do (a) leave-one-out (LOO) or leave-many-out (LMO) parameter-based internal validation; (b) external validation; (c) Y-scrambling or model applicability domain (AD) analysis in accordance with OECD requirements. The robustness of the GA-MLR-based QSAR model was previously assessed on the basis of how well the various statistical parameters perform on the respective starting value. Two QSAR models (1.1 and 1.2) consisting of univariate divided set and multivariate divided set models with excellent values on these parameters and best predictive capability were chosen for the analysis, but the rest of the QSAR models failed to fulfil some of these factors above-mention values and were omitted [35].

### 4.5. Molecular Docking

The protein data bank provided the ALK tyrosine kinase pdb file (pdb id-5fto, Resolution 1.7). For its X-ray resolution or sequel completeness, the pdb 5fto was carefully chosen. The optimized protein is appropriate for docking analysis. The protein preparation was carried by UCF chimera-1.16-win64 software (See Supplementary Materials for the detail procedure of protein preparation by chimera-1.16 software). Before the docking investigation, the natural ligand (Entrectinib) was removed. In the present study, the binding site for native ligand, namely the active site, has been studied. As a result, the compounds were docked between the active site, where the native ligand was originally bound along ALK tyrosine kinase, and the docking posture for the most active molecules 172 and 178 is shown below for convenience. The NRGSuite programme (PyMOL versions 1.2 and above) was used to do the molecular docking investigation. Because this is a free and open source software program, it may also be utilised as a PyMOL plugin [35]. It detects surface holes in a protein and uses them as target binding sites for docking simulations with the help of FlexAID [40]. It employs a genetic algorithm for function conformational search, model ligand and side-chain flexibility, and allows for covalent binding simulation. To achieve substantial performance with NRGSuite, the flexible–rigid docking approach was employed in conjunction with the following default settings: Because of the binding sites, the input technique is spherical (diameter: 17); the spacing on the three-dimensional grid is 0.385; facet band flexibility is no; ligand flexibility is yes; ligand posture is no; restrictions are no. Hetero groups-cloud molecules included; van der Walls permeability −0.1; solvent types-none; variation on chromosomes—1000; variation on generations—1000; fitness model-share; copy model-population boom; and variation on top complexes—5. After the docking process was validated, the molecule Entrectinib, a discovered tyrosine kinase inhibitor, was employed for validation.

### 4.6. MD Simulation Analysis

The virtual screening findings are utilised to evaluate the most active Molecule 178 with a docking score of −7.8 kcal/mol and Molecule 172 (−8.0 Kcal/mol) in molecular dynamics and simulation using the Schrodinger Desmond versus 2020.1 (MD simulation). The SPC (Simple factor charge) model was utilised to bind protein ligands using the docking complexes Molecule 178 and Molecule 172. In this system, the OPLS-2005 pressure subject and explicit solvent model with SPC water molecules were applied. To neutralise the charge, Na+ ions were added [41]. To imitate the physiological environment, 0.15 M NaCl alternatives are provided to the computer [42]. The Nose–Hoover chain coupling approach was employed to build up the NPT ensemble with temperature 300 K, leisure time of 1.0 ps, and pressure 1 bar, which was once as soon as maintained in all simulations. A 2 fs time step will be employed. The barostat approach with the Martyna–Tuckerman–Klein chain coupling scheme [43] was originally utilised for pressure control with a leisure time of 2 ps. The particle mesh Ewald technique [44] was used to calculate long-range electrostatic interactions with a radius of 9 for Coulomb interactions. The non-bonded forces were estimated using the RESPA integrator. The root mean square deviation (RMSD), root mean square fluctuation (RMSF), radius of gyration (Rg), and protein ligand interactions were assessed to have a check at the stability of the complex in MD simulations.

### 4.7. Molecular Mechanics Generalized Born and Surface Area (MMGBSA) Calculations

The binding free energy (Gbind) of docked complexes was determined using the molecular mechanics generalized born surface region (MM-GBSA) module in MD simulations comprising 5fto bonded with the most active molecule 178 and the molecule 172. (Schrodinger suite, LLC, New York, NY, USA, 2017-4). At around the same time, the binding free energy was estimated using the OPLS 2005 force field, the VSGB solvent model and rotamer search techniques [45]. Following the MD run, the MD trajectories frames were chosen at 10 ns intervals. The total free energy binding used to be calculated the usage of Equation (1):

∆Gbind = Gcomplex − (Gprotein + Gligand)(3)

where,

∆Gbind = binding free energy,
Gcomplex = free energy of the complex,
Gprotein = free energy of the target protein, and
Gligand = free energy of the ligand.

## 5. Conclusions

A cheminformatics technique was used effectively in the current investigation to predict ALK Tyrosine kinase inhibitory activity in order to uncover fundamental structural aspects important for anticancer activity. Two statistically robust univariate and four parametric QSAR models with exceptional external predictive capability were built, and the right number of molecular features were accurately positioned. The QSAR analysis effectively identified a combination based on previously unknown Pharmacophoric properties. The existence of fluorine atoms on the phenyl ring, as well as the presence of planar nitrogen atoms, must be retained in future drug design, coupled with some novel Pharmacophoric qualities such as rsa. The molecular descriptors identified in the developed QSAR models, such as the ratio of surface area (rsa), planar nitrogen within four bonds from the nitrogen atom, fluorine atom within five bonds from the non-ring oxygen atom, lipophilic atoms within two bonds from the ring carbon atoms, and so on, can potentially enhance the ALK Tyrosine kinase inhibition potency. QSAR and molecular docking studies have successfully identified certain significant Pharmacophoric traits, such as the presence of an extra carbon atom in molecule 172, which results in increased flexibility and rsa; comparison of molecule 176 with 178 reveals an interesting influence of the N_PlanN_4B and ringC_lipo_2Bon docking pose and activity profile; and reversal in docking conformation and differences in the binding affinities of compounds 176 and 178. Moreover, identification of the additional polar nitrogen in QSAR analysis responsible for increasing the polarity of the molecule. This observation reveals that the QSAR and docking results are completely consistent with one another. The molecular docking studies on the 172 and 178 with the ALK tyrosine kinase receptor revealed that these compounds anchored to the ALK tyrosine kinase along the orientation or position extremely close to co-crystallized ligand; Entrectinib that resulted from crystallographic analysis of the ALK tyrosine kinase protein including its actual ligand. As a result, the created QSAR models meet the threshold values for several statistical parameters required to get the accuracy and applicability of a QSAR model. As a result, the obtained QSAR models include an appropriate mix of quantitative and qualitative characteristics. The pharmacophoric properties found in QSAR models show tremendous potential for optimizing dataset compounds in accordance with more potent ALK tyrosine kinase inhibitors as anticancer leads. Furthermore, MD simulation and binding free energy analyses support the findings of the QSAR and molecular docking studies.

## Figures and Tables

**Table 1:** The statistical parameters connected with the fitting, double validation and Y-scrambling for models 1.1 and 1.2.

Statistical Parameters	Model-1.1 (Univariate Dividedset Model)	Model-1.2 (Multivariate DividedSet Model)
Fitting		
R^2^	0.699	0.86
R^2^_adj_	0.692	0.86
R^2^-R^2^_adj_	0.001	0.003
LOF	0.57	0.25
K_xx_	0.00	0.29
Delta K	0.83	0.14
RMSE_tr_	0.74	0.48
MAE_tr_	0.57	0.38
RSS_tr_	101.03	42.10
CCC_tr_	0.81	0.93
S	0.75	0.49
F	403.4	292.1
Internal Validation		
Q^2^_LOO_	0.68	0.86
R^2^-Q^2^_LOO_	0.005	0.007
RMSE_cv_	0.75	0.49
MAE_cv_	0.58	0.39
PRESS_cv_	102.9	44.6
CCC_cv_	0.81	0.92
Q^2^_LMO_	0.68	0.86
R^2^_Yscr_	0.005	0.02
RMSE AV_Yscr_	1.35	1.3
Q^2^_Yscr_	−0.01	0.03
External Validation		
RMSE_ext_	0.79	0.57
MAE_ext_	0.61	0.45
PRESS_ext_	27.8	14.3
R^2^_ext_	0.65	0.83
Q^2^-_F1_	0.64	0.82
Q^2^-_F2_	0.63	0.82
Q^2^-_F3_	0.65	0.81
CCC_ext_	0.80	0.90
r^2^m aver.	0.52	0.75
r^2^m delta	0.10	0.15
k’	0.99	0.99
K	0.98	0.99
Clos’	0.10	0.04
Clos	0.01	0.0

**Table 2:** Presentation of docking interactions of the compound 172.

Residue	Distance in Å	Type of Interaction	Type of Bonding	From	Nature	To	Nature	Angle DHA	Angle HAY
MET1199:H	2.17	Hydrogen Bond	Conventional Hydrogen Bond	MET1199	H-Donor	0:N	H-Acceptor	142.364	93.83
GLU1197	2.87	Hydrogen Bond	Conventional Hydrogen Bond	0:H	H-Donor	GLU1197	H-Acceptor	111.868	157.456
LEU1198	2.82	Hydrogen Bond	Carbon Hydrogen Bond	LEU1198	H-Donor	0:N	H-Acceptor	140.175	119.444
PHE1127	4.81	Hydrophobic	Pi-Pi Stacked	PHE1127	Pi-Orbitals	0	Pi-Orbitals		
VAL1130	4.28	Hydrophobic	Alkyl	VAL1130	Alkyl	0	Alkyl		
LEU1256	4.59	Hydrophobic	Alkyl	LEU1256	Alkyl	0	Alkyl		
PHE1127	4.48	Hydrophobic	Pi-Alkyl	PHE1127	Pi-Orbitals	0	Alkyl		
VAL1130	5.07	Hydrophobic	Pi-Alkyl	0	Pi-Orbitals	VAL1130	Alkyl		
ALA1148	3.54	Hydrophobic	Pi-Alkyl	0	Pi-Orbitals	ALA1148	Alkyl		
MET1199	5.43	Hydrophobic	Pi-Alkyl	0	Pi-Orbitals	MET1199	Alkyl		
LEU1256	4.62	Hydrophobic	Pi-Alkyl	0	Pi-Orbitals	LEU1256	Alkyl		
LEU1122	4.93	Hydrophobic	Pi-Alkyl	0	Pi-Orbitals	LEU1122	Alkyl		
VAL1130	3.94	Hydrophobic	Pi-Alkyl	0	Pi-Orbitals	VAL1130	Alkyl		
LEU1256	4.17	Hydrophobic	Pi-Alkyl	0	Pi-Orbitals	LEU1256	Alkyl		

**Table 3:** Presentation of docking interactions of the compound 178.

Residue	Distance in Å	Type of Interaction	Types of Bonding	From	Nature	To	Nature	Angle DHA	Angle HAY
HOH2080	2.87	Hydrogen Bond	Water Hydrogen Bond; Carbon Hydrogen Bond	0:H3	H-Donor	HOH2080	H-Acceptor	114.83	91.8
ASP1203	2.58	Hydrogen Bond	Carbon Hydrogen Bond	0:H1	H-Donor	ASP1203	H-Acceptor	119.9	102.5
ASP1203	2.70	Hydrogen Bond	Carbon Hydrogen Bond	0:H1	H-Donor	ASP1203	H-Acceptor	147.9	110.3
PHE1127	5.03	Hydrophobic	Pi-Pi Stacked	PHE1127	Pi-Orbitals	0	Pi-Orbitals		
PHE1127	3.82	Hydrophobic	Pi-Pi Stacked	0	Pi-Orbitals	PHE1127	Pi-Orbitals		
ALA1148	3.88	Hydrophobic	Alkyl	ALA1148	Alkyl	0:C	Alkyl		
LEU1256	4.66	Hydrophobic	Alkyl	0:C	Alkyl	LEU1256	Alkyl		
LEU1122	5.49	Hydrophobic	Alkyl	0:C	Alkyl	LEU1122	Alkyl		
LEU1198	5.22	Hydrophobic	Alkyl	0:C	Alkyl	LEU1198	Alkyl		
MET1199	5.47	Hydrophobic	Alkyl	0:C	Alkyl	MET1199	Alkyl		
PHE1127	4.51	Hydrophobic	Pi-Alkyl	PHE1127	Pi-Orbitals	0:C	Alkyl		
LEU1256	5.43	Hydrophobic	Pi-Alkyl	0	Pi-Orbitals	LEU1256	Alkyl		
VAL1130	4.14	Hydrophobic	Pi-Alkyl	0	Pi-Orbitals	VAL1130	Alkyl		
LEU1256	4.60	Hydrophobic	Pi-Alkyl	0	Pi-Orbitals	LEU1256	Alkyl		
LEU1122	3.84	Hydrophobic	Pi-Alkyl	0	Pi-Orbitals	LEU1122	Alkyl		

**Table 4:** Binding energy calculation of 172 and178 with ALK and non-bonded interaction energies from MMGBSA trajectories. (* indicates mean value of energy parameters).

Energies (kcal/mol) *	ALK-172	ALK-178
ΔGbind	−49.4 ± 4.2	−52.6 ± 3.0
ΔGbindLipo	−17.4 ± 0.6	−19.5 ± 1.5
ΔGbindvdW	−41.1 ± 3.2	−44.8 ± 3.1
ΔGbindCoulomb	−9.1 ± 3.5	−5.7 ± 2.2
ΔGbindHbond	−1.4 ± 0.6	−0.3 ± 0.2
ΔGbindSolvGB	19.7 ± 3.1	18.2 ± 1.8
ΔGbindCovalent	1.0 ± 0.9	1.3 ± 1.2

**Table 5:** Presentation of Serial number, ChEMBL ID, Smiles, pKi and Ki value of 10 most active and 10 least active molecules in the dataset as representative examples only.

Sn	CHEMBL ID	Smiles	pKi	Ki in nM
178	CHEMBL3286823	Cc1nn(C)c2c1-c1cnc(N)c(n1)O[C@H](C)c1cc(F)ccc1C(=O)N(C)C2	10	0.1
172	CHEMBL3286815	N#Cc1[nH]nc2c1-c1cnc(N)c(c1)OCc1cc(F)ccc1OCCC2	9.68	0.22
176	CHEMBL3286820	Cc1nn(C)c2c1-c1cnc(N)c(c1)O[C@H](C)c1cc(F)ccc1C(=O)N(C)C2	9.58	0.29
216	CHEMBL4286522	Cc1[nH][n+](C)c2c1-c1cnc(N)c(c1)O[C@H](C)c1cc(F)ccc1C(=O)N(C)C2	9.53	0.29
173	CHEMBL3286816	Cn1ncc2c1-c1cnc(N)c(c1)OCc1cc(F)ccc1OCCC2	9.42	0.38
161	CHEMBL3128064	Cc1nc(C(C)(C)O)sc1-c1cnc(N)c(O[C@H](C)c2cc(F)ccc2-n2nccn2)c1	9.39	0.4
181	CHEMBL3286832	C[C@H]1Oc2nc(cnc2N)-c2c(nc3ccc(C#N)cn23)CN(C)C(=O)c2ccc(F)cc21	9.25	0.56
174	CHEMBL3286818	C[C@H]1Oc2cc(cnc2N)-c2c(nn(C)c2C#N)CCOc2ccc(F)cc21	9.24	0.57
175	CHEMBL3286819	C[C@H]1Oc2cc(cnc2N)-c2c(nn(C)c2C#N)CCCOc2ccc(F)cc21	9.20	0.61
179	CHEMBL3286830	C[C@H]1Oc2cc(cnc2N)-c2c(nn(C)c2C#N)CN(C)C(=O)c2ccc(F)cc21	9.1	0.7
110	CHEMBL1995765	Nc1cc( = O)[nH]n1-c1ccccn1	4.5	31,622.7
13	CHEMBL1972934	Nc1ncnc2sccc12	4.4	39,810.7
39	CHEMBL1975212	Nc1ncnc2scc(-c3ccccc3)c12	4.4	39,810.7
48	CHEMBL1949855	O=c1[nH]cnc2c(Cl)cccc12	4.4	39,810.7
107	CHEMBL1994159	CC(=O)c1cccc(-c2ccc3nccn3n2)c1	4.3	50,118.7
129	CHEMBL2000879	c1ccc(C2CCc3[nH]ncc3C2)cc1	4.3	50,118.7
33	CHEMBL1971519	Nc1n[nH]c2cccc(-c3ccc(F)cc3)c12	4.2	63,095.7
34	CHEMBL1971534	NC(=O)c1cc2ccncc2s1	4.2	63,095.7
50	CHEMBL1975921	O=c1[nH]c2cc(Br)cnc2[nH]1	4.2	63,095.7
131	CHEMBL2007097	Nc1nc(=O)[nH]c2sccc12	4.1	79,432.8

## Data Availability

The data is available in the Supplementary Materials section.

## Supplementary Materials

The following supporting information can be downloaded at: https://www.mdpi.com/article/10.3390/molecules27154951/s1, Table S1: The SMILES notation for two hundred twenty four (224) ALK tyrosine kinase leads, along with their reported Ki and pKi values. Table S2: The values for selected molecular descriptors present in QSAR models. Table S3: Details regarding performance of Univariate Divided set model 1.1. containing Different graphs associated with model, Table S4: Details regarding performance of Multivariate divided set model comprising Different graphs associated with model 1.2. Statistical parameters for used for validation of QSAR models. File S5: Description of the performance parameters in QSARINS.

## Sample Availability

Samples of the compounds are available from the authors.
