# Supplementary material for: QSAR, Molecular Docking, MD Simulation and MMGBSA Calculations Approaches to Recognize Concealed Pharmacophoric Features Requisite for the Optimization of ALK Tyrosine Kinase Inhibitors as Anticancer Leads
Source: Molecules. 2022 Aug 3;27(15):4951. doi: 10.3390/molecules27154951 (PMC9370430; doi:10.3390/molecules27154951)
Supplement: Supplementary file 1 [file molecules-27-04951-s001.zip › File S5.pdf]

Description of the performance parameters in QSARINS

| Performance parameter | Calculated during | Formula | Description |
| --- | --- | --- | --- |
| $R^2$, $R^2_{ext}$ | training, external validation | $R^2 = 1 - \frac{\sum_{i=1}^{n}(y_i - \hat{y}_i)^2}{\sum_{i=1}^{n}(y_i - \bar{y})^2} = 1 - \frac{RSS}{TSS}$ | Explained variance; coefficient of determination, squareof the multiple correlation coefficient |
| $R^2_{adj.}$ | training | $R^2_{adj.} = R^2 - (1 - R^2) \times \frac{p}{n - p - 1}$ | $R^2$ corrected with the degree of freedom |
| $R^2 - R^2_{adj.}$ | training | see above | Difference of the two |
| LOF | training | $LOF = \frac{RSS}{n\left(1 - \frac{M + d(M-1)/2}{n}\right)^2}$ | Friedman lack of fit criteria [40]. M: total number of linearly independent bases in the model, d: degrees-of-freedom cost for each nonlinear basis function |
| $K_x$ | training | Based on PCA, see [41] for details | Inter-correlation among descriptors |

| | | | |
|---|---|---|---|
| *ΔK* | training | Based on PCA, see [41] for details | Difference of correlation among descriptors ($K_x$) and the descriptors plus responses ($K_{xy}$) |
| *RMSE* | training, int. val., ext. val. | $RMSE = \sqrt{\frac{\sum_{i=1}^{n}(y_i - \hat{y}_i)^2}{n}}$ | Root mean square error |
| *MAE* | training, int. val., ext. val. | $MAE = \frac{\sum_{i=1}^{n}\lvert y_i - \hat{y}_i\rvert}{n}$ | Mean absolute error |
| *RSS* | training | $RSS = \sum_{i=1}^{n}(y_i - \hat{y}_i)^2$ | Residual sum of squares |
| *CCC* | training, int. val., ext. val. | $CCC = \frac{2\sum_{i=1}^{n}(y_i - \bar{y})(\hat{y}_i - \hat{y})}{\sum_{i=1}^{n}(y_i - \bar{y})^2 + \sum_{i=1}^{n}(\hat{y}_i - \hat{y})^2 + n(\bar{y} - \hat{y})^2}$ | Coefficient of concordance, concordance correlationcoefficient [42,43] |
| *s* | training | $s = \sqrt{\frac{1}{N-1}\sum_{i=1}^{N}(y_i - \hat{y}_i)^2}$ | Standard error of the estimate |

| | | | |
|---|---|---|---|
| $F$ | training | $F = \left(\frac{\sum_{i=1}^{N}(\bar{y}-\hat{y}_i)^2}{p-1}\right) / \left(\frac{\sum_{i=1}^{N}(y_i-\hat{y}_i)^2}{n-p}\right)$ | Fisher value |

| | | | |
|---|---|---|---|
| $Q^2_{LOO}$ | internal validation | $Q^2_{LOO} = 1 - \frac{\sum_{i=1}^{n}(y_i-\hat{y}_{i/i})^2}{\sum_{i=1}^{n}(y_i-\bar{y})^2} = 1 - \frac{PRESS}{TSS}$ | Leave-one-out cross-validated square of the (multiple) correlation coefficient |
| $R^2 - Q^2_{LOO}$ | internal validation | see above | Difference of the two |
| $PRESS$ | internal, external validation | $PRESS = \sum_{i=1}^{n}(y_i-\hat{y}_{i/i})^2$ | Predicted residual sum of squares (either cross-validatedor calculated on the external set) |
| $Q^2_{LMO}$ | internal validation | $Q^2_{LMO} = 1 - \frac{\sum_{j=1}^{m}\sum_{i=1}^{n}(y_i-\hat{y}_{i/j})^2}{\sum_{i=1}^{n}(y_i-\bar{y})^2}$ | Leave-many-out cross-validated square of the (multiple) correlation coefficient |
| $R^2_{Y\text{-}SCRAMBLE}$ | internal validation | see above | $R^2$ of the training set with Y-scrambling [44] |

| | | | |
|---|---|---|---|
| $RMSE_{Avg,\ Y\text{-}SCRAMBLE}$ | internal validation | see above | Average RMSE with Y-scrambling [44] |
| $Q^2_{Y\text{-}SCRAMBLE}$ | internal validation | see above | $Q^2_{\mathrm{LOO}}$ of the training set with Y-scrambling [44] |

| | | | |
|---|---|---|---|
| $R^2_{RND\text{-}DESCR}$ | internal validation | see above | $R^2$ of the training set with randomized descriptors [44] |
| $Q^2_{RND\text{-}DESCR}$ | internal validation | see above | $Q^2_{\mathrm{LOO}}$ of the training set with randomized descriptors [44] |
| $R^2_{RND\text{-}RESP}$ | internal validation | see above | $R^2$ of the training set with randomized responses [44] |
| $Q^2_{RND\text{-}RESP}$ | internal validation | see above | $Q^2_{\mathrm{LOO}}$ of the training set with randomized responses [44] |
| $Q^2_{F1}$ | external validation | $Q^2_{F1} = 1 - \frac{\sum_{i=1}^{nEXT}(y_i - \hat{y}_i)^2}{\sum_{i=1}^{nEXT}(y_i - \bar{y}_{TR})^2}$ | Definition 1 in [35] for $Q^2$ of the external test set [40], TR: training set, EXT: external test set |

| | | | |
|---|---|---|---|
| $Q^2_{F2}$ | external validation | $Q^2_{F2} = 1 - \frac{\sum_{i=1}^{nEXT}(y_i - \hat{y}_i)^2}{\sum_{i=1}^{nEXT}(y_i - \bar{y}_{EXT})^2}$ | Definition 2 in [35] for $Q^2$ of the external test set [41], EXT: external test set |
| $Q^2_{F3}$ | external validation | $Q^2_{F3} = 1 - \frac{\sum_{i=1}^{nEXT}(y_i - \hat{y}_i)^2 / n_{EXT}}{\sum_{i=1}^{nTR}(y_i - \bar{y}_{TR})^2 / n_{TR}}$ | Definition 3 in [35] for $Q^2$ of the external test set [42], TR: training set, EXT: external test set |

| | | | |
|---|---|---|---|
| $r^2_m$ | external validation | $r^2_m = \frac{r^2_m + r'^2_m}{2}$ | Here, $r^2_m = R^2 \times (1 - \sqrt{R^2 - R^2_0})$, where $R_0^2$ is the squared correlation coefficient without intercept. $r'^2_m$ is the same as $r^2_m$, with the *x* and *y* axes exchanged. [43,44] |
| $\Delta r^2_m$ | external validation | $\Delta r^2_m = r^2_m - r'^2_m$ | See above. |

for *Case study 2* (c.f. figure 10)
